# Absence of Long-Term Protection in Domestic Pigs Immunized with Attenuated African Swine Fever Virus Isolate OURT88/3 or BeninΔMGF Correlates with Increased Levels of Regulatory T Cells and Interleukin-10

**DOI:** 10.1128/JVI.00350-20

**Published:** 2020-07-01

**Authors:** Pedro J. Sánchez-Cordón, Tamara Jabbar, Dave Chapman, Linda K. Dixon, María Montoya

**Affiliations:** a The Pirbright Institute, Woking, Surrey, United Kingdom; b Centro de Investigaciones Biológicas (CIB), CSIC, Madrid, Spain; University of Illinois at Urbana Champaign

**Keywords:** African swine fever virus, interleukin-10, attenuated vaccines, long-term immunity, regulatory T cells

## Abstract

Following short immunization protocols, naturally attenuated African swine fever virus (ASFV) isolate OURT88/3 and deletion mutant BeninΔMGF have previously been shown to induce high percentages of protection in domestic pigs against challenge with virulent virus. The results obtained in the present study show that a single intramuscular immunization of domestic pigs with OURT88/3 or BeninΔMGF followed by a challenge with the virulent Benin 97/1 isolate at day 130 postimmunization did not trigger the mechanisms necessary to generate immunological memory able to induce long-term protection against disease. All pigs developed acute forms of acute swine fever (ASF). Gamma interferon-producing cells peaked at day 24 postimmunization, declining thereafter. Surprisingly, the levels of regulatory T cells (Tregs) and interleukin-10 (IL-10) were elevated at the end of the experiment, suggesting that regulatory components of the immune system may inhibit effective protection.

**IMPORTANCE** The duration of immunity for any vaccine candidate is crucial. In the case of African swine fever virus vaccine candidates, this issue has received little attention. Attenuated viruses have proven protective following short immunization protocols in which pigs were challenged a few weeks after the first immunization. Here, the duration of immunity and the immune responses induced over a duration of 130 days were studied during prechallenge and after challenge of pigs immunized with the naturally attenuated isolate OURT88/3 and an attenuated gene-deleted isolate, BeninΔMGF. After a single intramuscular immunization of domestic pigs with the OURT88/3 isolate or BeninΔMGF virus, animals were not protected against challenge with the virulent Benin 97/1 ASFV genotype I isolate at day 130 postimmunization. The levels of regulatory T cells and IL-10 were elevated at the end of the experiment, suggesting that regulatory components of the immune system may inhibit effective protection.

## Abstract (precis)

The duration of immunity for any vaccine candidate is crucial. In the case of African swine fever virus vaccine candidates, this issue has received little attention. Attenuated viruses have proven protective following short immunization protocols in which pigs were challenged a few weeks after the first immunization. Here, the duration of immunity and the immune responses induced over a duration of 130 days were studied during prechallenge and after challenge of pigs immunized with the naturally attenuated isolate OURT88/3 and an attenuated gene-deleted isolate, BeninΔMGF. After a single intramuscular immunization of domestic pigs with the OURT88/3 isolate or BeninΔMGF virus, animals were not protected against challenge with the virulent Benin 97/1 ASFV genotype I isolate at day 130 postimmunization. The levels of regulatory T cells and IL-10 were elevated at the end of the experiment, suggesting that regulatory components of the immune system may inhibit effective protection.

## INTRODUCTION

African swine fever (ASF) is a frequently fatal hemorrhagic fever of domestic pigs and wild boar that has a high socioeconomic impact in affected countries. The causative agent, African swine fever virus (ASFV), is the only member of the *Asfarviridae* family, genus *Asfivirus* (1). ASFV is a large cytoplasmic DNA virus whose genome encodes up to 167 proteins and whose viral particles are complex, containing 69 virus proteins in several layers (2).

ASF is endemic or causes sporadic disease outbreaks in most of sub-Saharan Africa and in Sardinia, Italy. A transcontinental spread of genotype II ASFV from southeast Africa into Georgia occurred in 2007. Since then, ASF has spread to the Russian Federation and Eastern Europe and then into Western Europe, reaching Belgium in September 2018 (3). In August 2018, the disease reached the world’s largest pig producer, China (4), spreading out of control to several countries in Southeast Asia until reaching in September 2019 the very doors of the Australian continent (5). As a result, millions of pigs have died or have been culled. Combined with production losses due to the culling of breeding sows, the Chinese pig herd was reported to have been reduced by 37% in September 2019 compared to its size in the previous year (6). This represents about a quarter of the global pig herd, which has resulted in shortages and increased prices of pork, particularly in China. The lack of a vaccine limits control, and this is further complicated by the presence of wildlife reservoirs, including wild boar in Europe and Asia, other wild suids (7), and soft ticks of the *Ornithodoros* genus in some regions (8).

Early research toward vaccine development established that inactivated virions of ASFV did not protect pigs against challenge with virulent virus (9). This concept was confirmed in a recent study in which modern adjuvants were administered with inactivated virions (10). The failure of inactivated virions to induce protection is not surprising, given evidence that cellular immunity is required for protection (11). In contrast, it is well established that pigs which recover from infection with lower-virulence isolates can be protected against challenge with virulent isolates (12, 13).

In early experiments, ASFV isolates attenuated by sequential passage in cell cultures were also able to induce protection in domestic pigs, as determined by challenge with related virulent isolates (14). The naturally attenuated ASFV genotype I isolates NH/P68 and OURT88/3 have been used experimentally to understand the mechanisms of protection against challenge. Protection induced by OURT88/3 requires cellular immunity, since it was abrogated when CD8^+^ cells were depleted using specific antibodies (11). The protection against homologous and heterologous challenge induced by OURT88/3 correlated with the induction of high numbers of ASFV-specific gamma interferon (IFN-γ)-producing lymphocytes (15). However, such a correlation was not always observed (16). A key role for innate immunity in protection was also suggested, since the protection induced by the NH/P68 isolate correlated with an early increase in the numbers of NK cells (17). The role of antibodies in protection has also been suggested, since the passive transfer of sera from pigs recovered from infection to naive pigs conferred partial protection against challenge (18, 19).

Targeted gene deletions have been pursued as a strategy to produce safe and effective live attenuated vaccine candidates. Deletion of genes coding for IFN-inhibitory proteins can result in virus attenuation and the induction of protection. For example, the deletion of several members of multigene families (MGF; MGF 360 and MGF 505/530) from virulent isolates resulted in virus attenuation and the induction of protection against homologous challenge (20–22).

The duration of immunity by any vaccination protocol is a key factor for evaluating its potential. In the case of ASFV vaccine candidates, this issue has received little, if any, attention. In order to optimize immunization protocols with OURT88/3 and BeninΔMGF, different routes of inoculation (intramuscular and intranasal) and different doses (from 10^2^ to 10^5^ 50% tissue culture infective doses [TCID_50_]) were tested in previous experiments. Single immunizations with OURT88/3 by a protocol comparable to the immunization protocol used in the present study (10^4^ TCID_50_ by the intramuscular route) induced percentages of protection that ranged from 70% (23) to 100% (14) when animals were challenged shortly (21 days) after the immunization. Single immunizations with BeninΔMGF have not been tested before. However, even lower doses (10^2^ and 10^3^ TCID_50_) administered twice by the intramuscular route over an interval of 3 weeks were able to induce up to 100% protection (22, 24) when animals were challenged shortly (18 to 21 days) after the boost immunization. On the basis of these results and with the objective of performing a comparative evaluation, both vaccine candidates were selected to test in parallel their capacity to induce long-term protection after a single dose (10^4^ TCID_50_) was administered by the intramuscular route, considered the most feasible and safest route for immunizations. In the present study, we investigated the immune responses induced over a duration of 130 days prechallenge and after challenge of pigs immunized with the naturally attenuated isolate OURT88/3 and an attenuated gene-deleted isolate, BeninΔMGF. The naturally attenuated isolate OURT88/3 has a large deletion of genes coding for six members of MGF 360 and two members of MGF 505-R, in addition to an interruption of genes coding for a CD2-like protein and C-type lectin (25). The BeninΔMGF virus has the same MGF genes deleted and interruptions in one additional member of each MGF (22).

The results from the current study show that after a single intramuscular immunization of domestic pigs with the OURT88/3 isolate or BeninΔMGF, animals were not protected against challenge with the virulent Benin 97/1 ASFV genotype I isolate at day 130 postimmunization, although pigs immunized with BeninΔMGF survived longer. IFN-γ-producing cells peaked at day 24 postimmunization, declining thereafter. All pigs were euthanized at between 5 and 11 days postchallenge (p.c.) and showed clinical signs and ASF lesions equivalent to those described in nonimmunized control pigs. Surprisingly, the levels of regulatory T cells (Tregs) and interleukin-10 (IL-10) were elevated at the end of the experiment, suggesting that regulatory components of the immune system could inhibit effective protection. This study showed that a single dose of attenuated ASFV strains OURT88/3 and BeninΔMGF does not provide the means for immunological memory able to induce full long-term protection against challenge. It remains to be seen whether protection is achieved in the long term after boosting.

## RESULTS

### Clinical scores after immunization and challenge of pigs.

**FIG 1:**
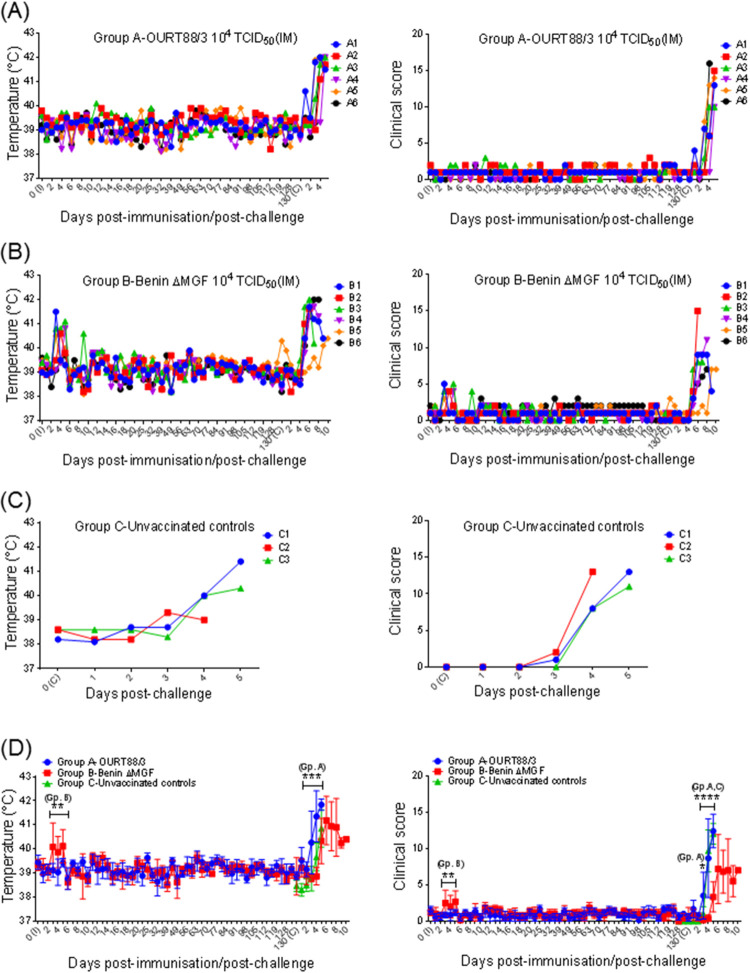
(A to C) Kinetics of rectal temperatures and clinical scores (means ± SD) of pigs immunized with OURT88/3 (A) or BeninΔMGF (B) or unvaccinated controls (C). (D) Rectal temperatures and clinical scores were assessed at different days after immunization and challenge of the pigs. Statistical analysis was carried out using a two-way analysis of variance. Asterisks indicate statistically significant differences between experimental groups (*, *P* < 0.05; **, *P* < 0.01; ***, *P* < 0.001; ****, *P* < 0.0001). I, immunization day (*x* axis); C, challenge day (*x* axis); Gp, group; IM, intramuscular.

OURT88/3-immunized pigs (group A) did not show any significant clinical signs during the 130 days postimmunization (p.i.) until challenge. However, with the exception of pig B6, a transient increase in rectal temperature above 40.5°C for 1 or 3 days was observed in all BeninΔMGF-immunized pigs (group B) between days 3 and 5 p.i. Apart from an increase in temperature, no other clinical signs were observed (Fig. 1A and B). Statistical analysis confirmed a significant increase in average temperatures and clinical scores in pigs in group B between days 3 and 5 p.i. (Fig. 1D).

**FIG 2:**
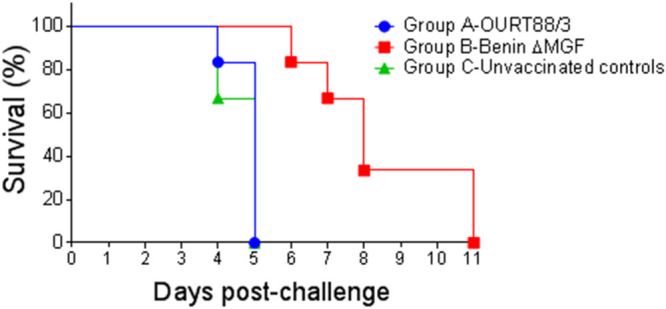
Percentage of surviving pigs after challenge. Groups of pigs (*n* = 6) were immunized by the intramuscular (IM) route with a single dose (10^4^ TCID_50_) of deletion mutant BeninΔMGF or low-virulent ASFV isolate OURT88/3. At day 130 postimmunization (p.i.), all immunized pigs, in parallel with three control nonimmune pigs, were challenged intramuscularly with 1 ml containing 10^4^ TCID_50_ of the genotype I virulent ASFV isolate Benin 97/1. Days postchallenge are shown on the *x* axis, and the percentage of protected pigs is shown on the *y* axis.

After challenge, all pigs developed clinical signs typical of acute ASF and were euthanized after reaching a moderate-severity humane endpoint (Fig. 1A to C). Statistical analysis showed a significant increase in the temperatures and clinical scores of the pigs in group A with respect to those of pigs in group B between days 3 and 5 postchallenge (p.c.), as well as a significant increase in the clinical scores of pigs in the control group (group C) compared to those of pigs in group B at days 4 and 5 p.c. (Fig. 1D). Pigs in group A were euthanized at the same time as control pigs in group C, between days 4 and 5 p.c. Pigs in group B survived for a longer period and were euthanized at days 6 and 7 p.c. (pigs B2 and B3, respectively), day 8 p.c. (pigs B4 and B6), and day 11 p.c. (pigs B1 and B5) (Fig. 2).

All pigs were assessed during necropsies. Both nonimmunized control pigs and nonprotected immunized pigs showed gross lesions characteristic of acute forms of ASF.

### Levels of virus genome and infectious virus in blood samples.

**FIG 3:**
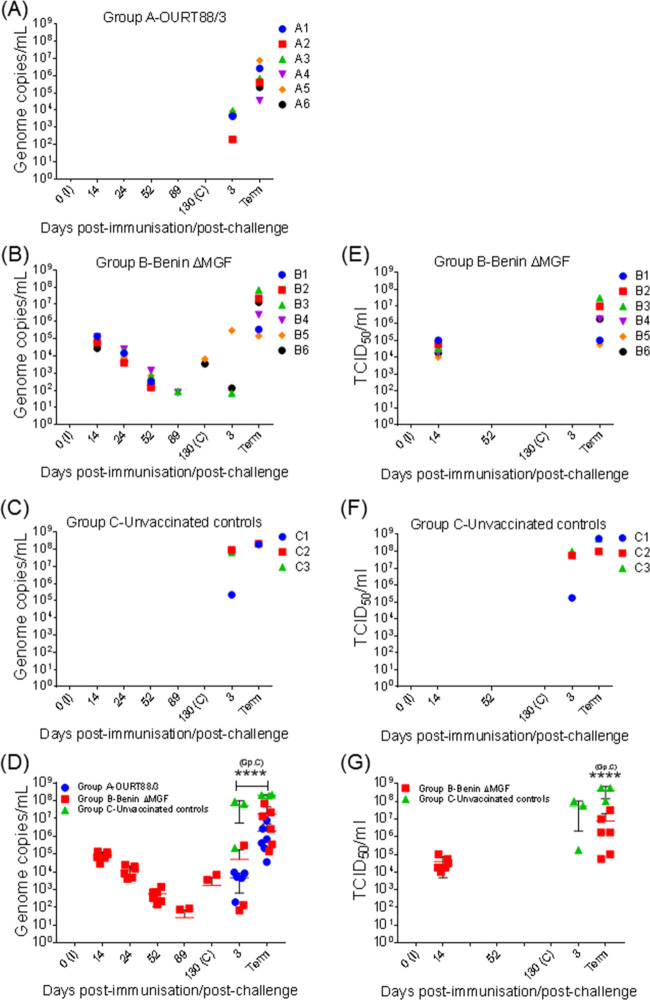
Kinetics of viremia levels (A to C) and infectious virus levels (E and F) in blood samples taken throughout the experiment. (D) Number of ASFV genome copies (mean ± SD) in blood samples. EDTA-anticoagulated blood samples were taken at different days after immunization (I) and challenge (C) and at termination (Term). Viral genome copies were determined individually by qPCR and are represented as the total number of genome copies per milliliter. (G) Level of infectious virus (mean ± SD) in blood samples. The amount of infectious virus was determined individually in blood samples from pigs immunized with BeninΔMGF and nonimmunized control pigs after immunization and challenge and at termination. Virus titers are presented individually as the amount of virus infecting 50% of the macrophage cultures (number of TCID_50_ per milliliter). Statistical analysis was carried out using a two-way analysis of variance. Asterisks indicate statistically significant differences between experimental groups (****, *P* < 0.0001).

Virus genome levels and the presence of infectious virus were evaluated in blood samples taken throughout the experiment by quantitative PCR (qPCR) and by titrating infectious virus particles. As expected from previous experiments (15, 23), no virus genome was detected in blood postimmunization from any of the pigs included in group A immunized with OURT88/3 (Fig. 3A).

Similar to previous results (22, 24), the virus genome was detected in blood samples from all animals immunized with BeninΔMGF (group B) at days 14, 24, and 52 p.i. Viremia levels decreased progressively from day 14 p.i. in all animals until reaching very low levels (less than 10^2^ copies/ml for pigs B3 and B4) or nondetectable levels at day 89 p.i. At day 130 p.i., low levels of virus (10^3^ copies/ml) were detected only in pigs B5 and B6 (Fig. 3B). Despite the presence of detectable levels of the virus genome, the pigs did not display any clinical signs during this stage of the experiment.

After challenge, low-to-moderate levels of viral genome were detected from day 3 p.c. in most pigs belonging to group A and some pigs belonging to group B. At termination, high levels of virus genome, which ranged from 6.92 × 10^7^ copies/ml for pig B3 to 3.59 × 10^4^ copies/ml for pig A4, were detected in all pigs immunized (Fig. 3A and B).

In all nonimmunized control pigs (group C), high levels of viremia were detected from day 3 p.c. (between 10^5^ and 10^7^ copies/ml), reaching the highest viremia levels at termination (up to 2.22 × 10^8^ copies/ml in pig C3). The levels of the virus genome in blood collected postchallenge from pigs in group C were consistent with those detected in immunized pigs (Fig. 3C). Statistical analysis showed that the means of the viremia levels were significantly lower (*P* < 0.0001) both at day 3 p.c. and at termination between both the group A and B pigs and the control pigs in group C (Fig. 3D).

Since in previous experiments with another attenuated deletion mutant (BeninΔDP148R) the virus genome persisted for longer periods in blood than infectious virus did, a phenomenon attributed to the virus’s ability to bind to the surface of red blood cells mediated by the CD2v protein rather than the presence of replicating virus (26), the levels of infectious virus in blood from pigs immunized with BeninΔMGF were also evaluated. Infectious virus was detected at levels of 10^4^ to 10^5^ TCID_50_ in blood from all animals at day 14 p.i. but not at day 52 or 130 p.i. After challenge, infectious virus was not detected at day 3 p.c., but levels of 10^5^ to 10^7^ TCID_50_ were detected at termination. In control nonimmune pigs, infectious virus was detected at day 3 p.c. (10^5^ to 10^8^ TCID_50_) and at termination (10^8^ to 10^9^ TCID_50_) (Fig. 3E and F). Therefore, in pigs immunized with BeninΔMGF, the levels of infectious virus at day 14 p.i. were similar to the virus genome levels. After this date, the virus genome but not infectious virus was detected over a long time period. This is probably due to virus binding to red blood cells and gradually losing infectivity over time, as we observed previously (26). This binding is mediated by the virus CD2v/EP402R protein, which is functional in the BeninΔMGF genome but not in the OURT88/3 isolate genome. After challenge, the levels of infectious virus detected at termination in the control group were significantly higher than those detected in the group of pigs immunized with BeninΔMGF (Fig. 3G). This indicates that the pigs in the group immunized with BeninΔMGF were partially protected since they had reduced virus replication after challenge and survived longer than the control pigs.

### ASFV-specific antibody response after immunization and challenge.

**FIG 4:**
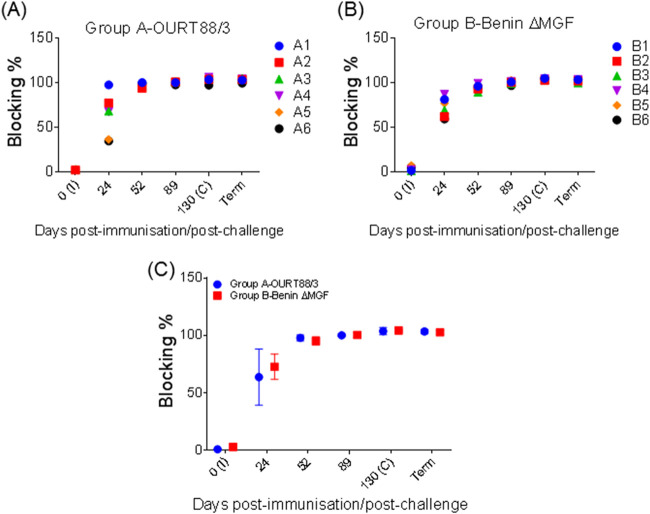
(A and B) Kinetics of ASFV-specific antibodies in serum samples taken at different days throughout the experiment from pigs immunized with OURT88/3 (A) or BeninΔMGF (B). (C) Levels of ASFV-specific antibodies (mean ± SD) in serum samples, measured by blocking ELISA. Serum samples with a blocking percentage of >50% were considered positive. Serum samples were taken at different days after immunization (I) and challenge (C) and at termination (Term). Statistical analysis was carried out using a two-way analysis of variance.

The serum levels of ASFV-specific antibodies were assessed at days 0, 24, 52, 89, and 130 p.i. and at termination. In OURT88/3-immunized pigs (group A), anti-VP72 responses were detected at day 24 p.i. and reached a plateau in all pigs by day 52 p.i., which was maintained throughout the experiment. Similar results were obtained in BeninΔMGF-immunized pigs (group B) (Fig. 4A and B). Significant differences were not found between the immunized pig groups (Fig. 4C). ASFV-specific antibodies were not detected in control pigs (group C) euthanized at days 4 and 5 p.c.

### Evaluation of IFN-α concentrations in serum samples.

**FIG 5:**
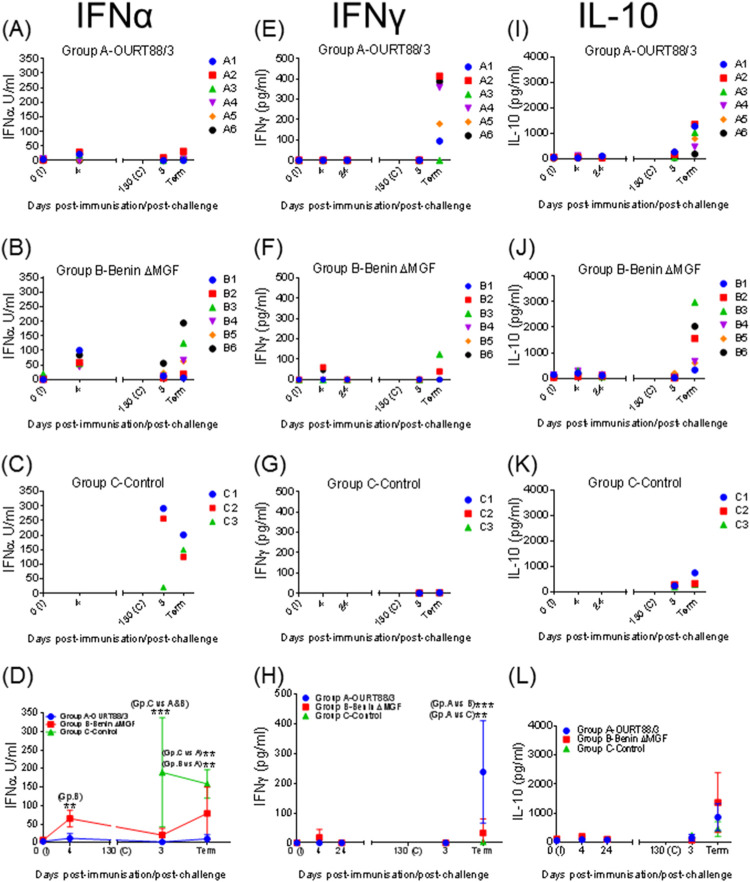
Kinetics of serum concentrations of IFN-α (A to C), IFN-γ (E to G), and IL-10 (I to K) in serum samples taken at different days throughout the experiment from pigs immunized with OURT88/3 or BeninΔMGF or unvaccinated controls and assayed by ELISA. The serum concentrations (mean ± SD) of IFN-α (D), IFN-γ (H), and IL-10 (L) are also shown. Serum samples were taken at different days after immunization (I) and challenge (C) and at termination (Term). Statistical analysis was carried out using a two-way analysis of variance. Asterisks indicate statistically significant differences between experimental groups (**, *P* < 0.01; ***, *P* < 0.001).

The levels of IFN-α, as a measure of the innate immune response, were evaluated by enzyme-linked immunosorbent assay (ELISA) in serum samples taken before and after immunization (day 0 and 4 p.i.), after challenge (day 3 p.c.), and at termination. All pigs immunized with BeninΔMGF showed an increase in IFN-α levels at day 4 p.i., whereas only some pigs vaccinated with OURT88/3 (pigs A1 and A2) displayed a mild increase (Fig. 5A and B). Significant differences between the two groups (*P* < 0.007) were observed at that time (Fig. 5D). After challenge, immunized and control pigs displayed high levels of IFN-α before termination (Fig. 5A to C). IFN-α concentrations were significantly higher in control pigs and pigs immunized with BeninΔMGF than in pigs immunized with OURT88/3 (Fig. 5D). IFN-α levels were especially high at termination in some pigs immunized with BeninΔMGF that were euthanized at late times of between days 7 and 11 p.c. (pigs B3, B4, B5, and B6) (Fig. 5B).

### Evaluation of IFN-γ secretion by ELISA and ELISpot assay.

The levels of IFN-γ, as a measure of induction of an adaptive immune response, in serum samples taken before and after immunization (days 0, 4, and 24 p.i.), after challenge (day 3 p.c.), and at termination were evaluated by ELISA. After immunization, no significant changes in IFN-γ levels were observed in pigs immunized with OURT88/3 or BeninΔMGF (Fig. 5E and F), except for 2 pigs immunized with BeninΔMGF (pigs B2 and B6), which showed a mild increase in IFN-γ concentrations at day 4 p.i. However, high levels of IFN-γ were detected in both groups of immunized pigs after challenge. IFN-γ concentrations were significantly higher at termination (*P < *0.001) in the group of pigs immunized with OURT88/3 than in the group of pigs immunized with BeninΔMGF and the control group (Fig. 5H). Only some pigs immunized with BeninΔMGF (pigs B2, B3, and B4) showed detectable IFN-γ levels at termination, while changes in IFN-γ concentrations were not observed in control pigs (Fig. 5F and G).

**FIG 6:**
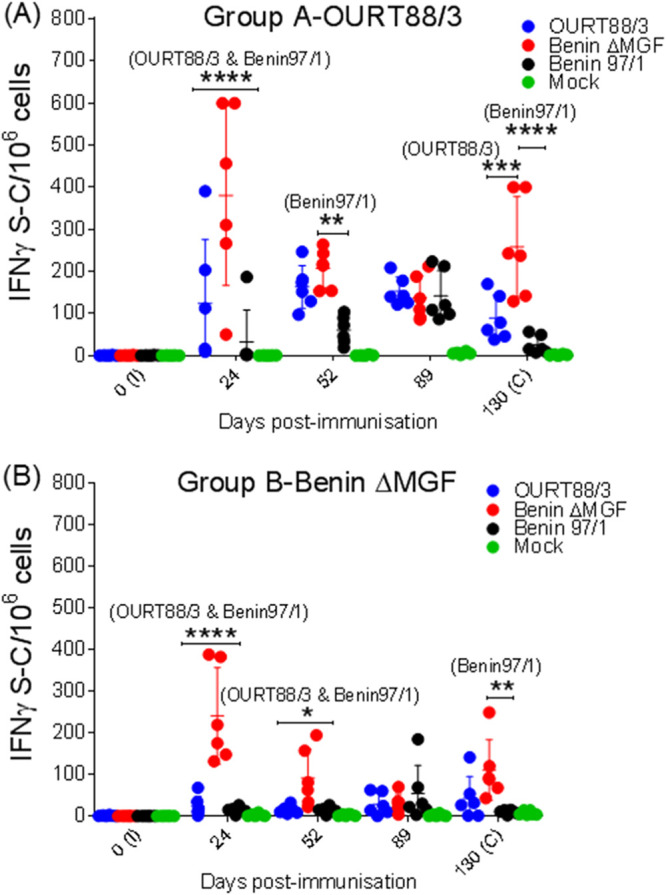
(A and B) Immune responses in vaccinated pigs. The number of IFN-γ-producing cells was determined by ELISpot assays using PBMCs purified from pigs immunized with OURT88/3 (A) or BeninΔMGF (B) that were stimulated with OURT88/3, BeninΔMGF, and Benin 97/1, medium (mock inoculum), and phytohemagglutinin (PHA; data not shown). The results show the numbers (mean ± SD) of IFN-γ-secreting cells (S-C) per 10^6^ cells (*y* axis). As expected, no specific IFN-γ-secreting cells were detected when mock stimulant was used in any animal at any time point, and the results for positive controls were in the range of 200 to 300 spots/10^6^ cells (data not shown). Statistical analysis was carried out using a two-way analysis of variance. Asterisks indicate statistically significant differences between experimental groups (*, *P* < 0.05; **, *P* < 0.01; ***, *P* < 0.001; ****, *P* < 0.0001). I, immunization day; C, challenge day (*x* axis).

In addition, the number of specific IFN-γ-producing cells induced at different days postimmunization was analyzed by enzyme-linked immunosorbent spot (ELISpot) assay following stimulation of peripheral blood mononuclear cells (PBMCs) with ASFV isolate Benin 97/1, OURT88/3, or BeninΔMGF, to determine whether cells with different specificities were generated and maintained over time. A negative-control stimulus (mock inoculum) and a positive-control stimulus (phytohemagglutinin [PHA]) were also used. As expected, no specific IFN-γ-secreting cells were detected when mock stimulant was used in any animal at any time point, and positive controls were in the range of 200 to 300 spots/10^6^ cells (data not shown). In pigs vaccinated with OURT88/3 (group A), an increase in the number of IFN-γ-secreting cells was observed mainly following stimulation with attenuated strain OURT88/3 or BeninΔMGF. The number of IFN-γ-secreting cells was significantly higher after stimulation with BeninΔMGF than after stimulation with OURT88/3 or Benin 97/1 at days 24, 52, and 130 p.i. Throughout the experiment, the lowest numbers of IFN-γ-secreting cells were observed after stimulation with the virulent Benin 97/1 strain (Fig. 6A). In pigs vaccinated with BeninΔMGF (group B), BeninΔMGF stimulation also induced an increase in IFN-γ-secreting cells that was significantly higher at days 24, 52, and 130 p.i. than the response induced by stimulation with OURT88/3 or Benin 97/1 (Fig. 6B).

### Identification of cell subsets secreting IFN-γ.

Remaining questions were which cells were responsible for secreting IFN-γ after priming immune responses with each attenuated virus and whether they differed when using different viruses. PBMCs taken from immunized animals throughout the experiment were stimulated *in vitro* and analyzed by flow cytometry, combining staining for intracellular IFN-γ with phenotyping of T cells (CD3^+^). Stimulation was carried out using ASFV isolate Benin 97/1, OURT88/3, or BeninΔMGF. A negative-control stimulus (mock inoculum) and a positive-control stimulus (PMA-ionomycin [ION]) were also used. When a mitogen was used as a positive control, along with NK cells, the majority of CD3^+^ cells producing IFN-γ were γδ-T cells (CD4^+^ T-cell receptor γδ positive [TCRγδ^+^]), memory cells (CD4α^+^ CD8α^+^), and cytotoxic T cells (CD4α^−^ CD8α^+^) (data not shown).

**FIG 7:**
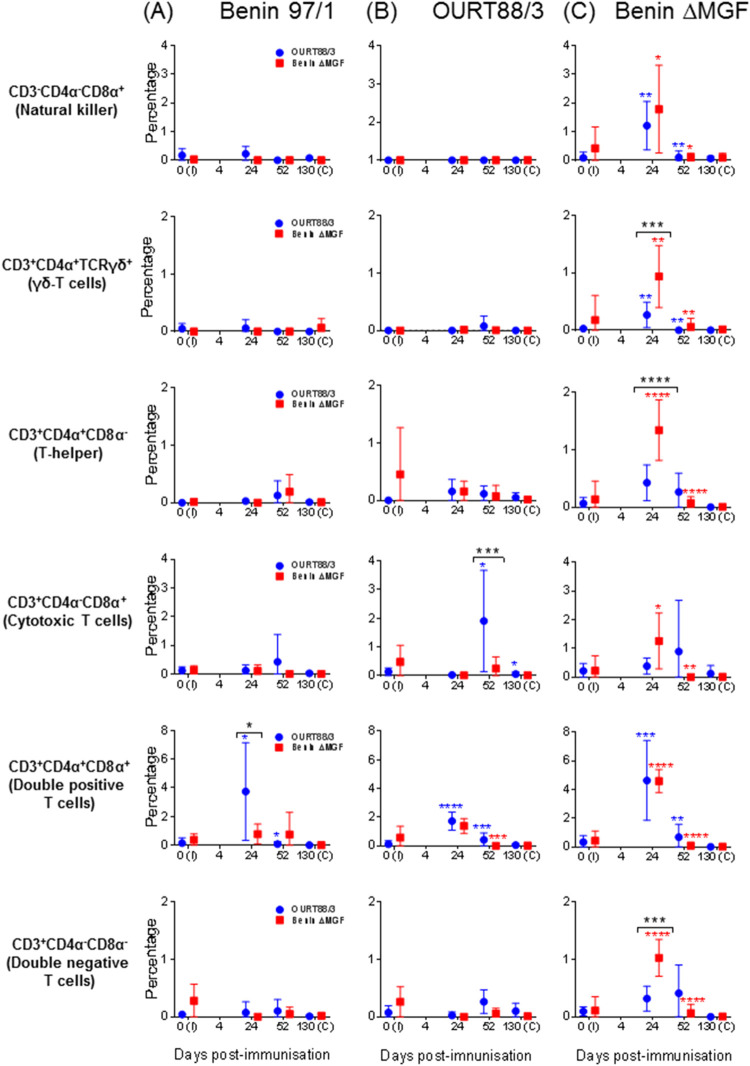
Changes in the percentages of IFN-γ-positive T cells. PBMCs obtained from pigs immunized with OURT88/3 or BeninΔMGF throughout the experiment were stimulated with medium (mock; data not shown), PMA-ION (data not shown), Benin 97/1 (A), OURT88/3 (B), or BeninΔMGF (C). The results show the percentages (mean ± SD) of IFN-γ-positive T-cell subsets (*y* axis) assessed by flow cytometry. Red and blue asterisks indicate statistically significant differences (one-way analysis of variance) from the previous date analyzed within each experimental group. Black asterisks indicate statistically significant differences (two-way analysis of variance) between the experimental groups (*, *P* < 0.05; **, *P* < 0.01; ***, *P* < 0.001; ****, *P* < 0.0001). I, immunization day; C, challenge day (*x* axis).

Stimulation with Benin 97/1 or OURT88/3 barely induced changes in the level of IFN-γ production by different cell subsets evaluated in immunized pigs in either group A (immunized with OURT88/3) or group B (immunized with BeninΔMGF) (Fig. 7). Significant differences between groups A and B in the percentage of cytotoxic T cells secreting IFN-γ after stimulation with OURT88/3 at day 52 p.i. (Fig. 7B) and in the percentage of double-positive cells secreting IFN-γ at day 24 p.i. after stimulation with Benin 97/1 (Fig. 7A) were highlighted. On the other hand, stimulation with BeninΔMGF induced a transient increase in the percentage of IFN-γ-secreting cells, including most subsets evaluated, in both groups of immunized pigs. Significant differences in the percentage of γδ-T cells, T-helper cells, and double-negative cells secreting IFN-γ were observed between group A and group B at day 24 p.i. In addition, a significant transient increase in the percentage of NK cells, cytotoxic T cells, and memory cells secreting IFN-γ that peaked at day 24 p.i. was also observed in both groups of immunized pigs (Fig. 7C).

### Evolution of cell subsets in PBMCs after immunization.

**FIG 8:**
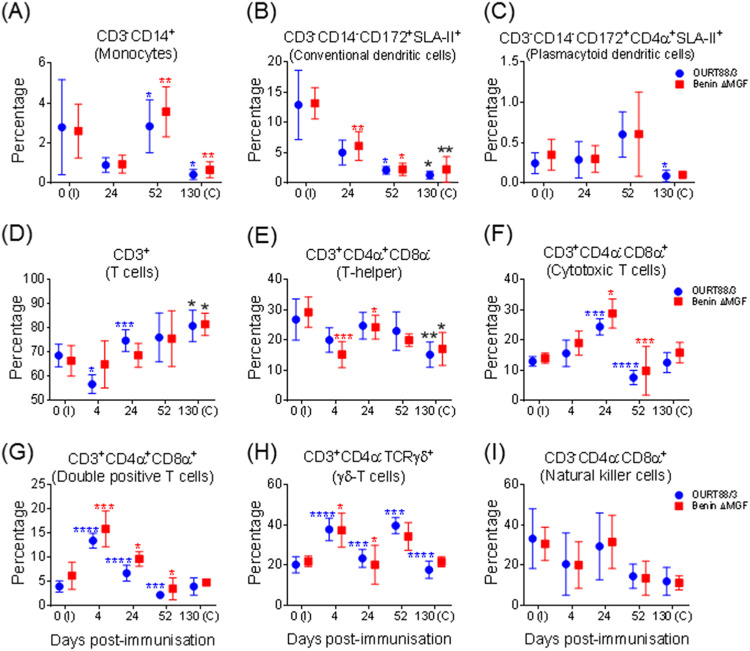
Immunophenotyping of PBMCs obtained from pigs immunized with OURT88/3 or BeninΔMGF by flow cytometry. Results show the percentages (mean ± SD) and kinetics of monocytes (A), dendritic cell subsets (B to C), and T-cell subsets (D to I) throughout the experiment. Red and blue asterisks indicate statistically significant differences (one-way analysis of variance) from the previous date analyzed within each experimental group. Black asterisks indicate statistically significant differences (one-way analysis of variance) between preimmunization percentages at day 0 p.i. and prechallenge percentages at day 130 p.i. within each experimental group (*, *P* < 0.05; **, *P* < 0.01; ***, *P* < 0.001; ****, *P* < 0.0001). I, immunization day; C, challenge day (*x* axis).

One plausible explanation for the low number of IFN-γ-producing cells after vaccination could be that PBMC subsets may suffer changes in their numbers during immunization. To test this hypothesis, cell subsets in PBMCs were analyzed at different postimmunization dates before challenge. As shown in Fig. 8, CD3^−^ CD14^+^ cells (Fig. 8A) fluctuated after immunization of pigs with BeninΔMGF or OURT88/3, exhibiting a statistically significant increase at day 52 p.i. compared to that at day 24 p.i., followed by a significant decrease at day 130 p.i., with the values being close to the preimmunization percentages. As for conventional dendritic cells (DCs; CD3^−^ CD14^−^ CD172^+^ swine leukocyte antigen II positive [SLA-II^+^]), there was a constant and significant decrease in the percentage of those cells during the period studied, reaching at day 130 p.i. values significantly lower than the percentage detected before immunization (Fig. 8B). On the contrary, plasmacytoid DCs (pDCs; CD3^−^ CD14^−^ CD172^+^ CD4α^+^ SLA-II^+^) exhibited a nonsignificant increase at day 52 p.i., which decreased at day 130 p.i., reaching normal preimmunization percentages (Fig. 8C).

Analysis of T cells (CD3^+^) showed that, after an initial mild decrease in PBMCs at day 4 p.i., the T-cell population recovered or increased its levels, peaking at day 130 p.i., with the levels being significantly higher than the values detected before immunization (Fig. 8D). Among T-cell subsets, T-helper cells (CD3^+^ CD4α^+^ CD8α^−^) showed a progressive decrease, reaching at day 130 p.i. values significantly lower than those detected before immunization (Fig. 8E). The numbers of cytotoxic T cells (CD3^+^ CD4α^−^ CD8α^+^) fluctuated along the period studied, showing a significant increase at day 24 p.i., followed by a significant decrease at day 52 p.i., along with the recovery of preimmunization percentages at day 130 p.i. (Fig. 8F). The double-positive cell subset (CD3^+^ CD4α^+^ CD8α^+^) significantly increased at day 4 p.i., followed by a dramatic decrease up to day 52 p.i., along with a mild recovery of levels at day 130 p.i., when percentages close to preimmunization values were observed (Fig. 8G). γδ-T cells also showed fluctuations along the period evaluated, with peaks at days 4 and 52 p.i., returning to normal percentages at day 130 p.i. (Fig. 8H). After initial fluctuations, NK cells showed a progressive, but not significant, decrease in percentage from day 24 p.i. (Fig. 8I).

Despite the fluctuations observed, the statistical comparative study between the two experimental groups did not show any significant differences regarding the evolution of any of the cell populations analyzed at any time after immunization.

### Changes in Tregs after immunization.

**FIG 9:**
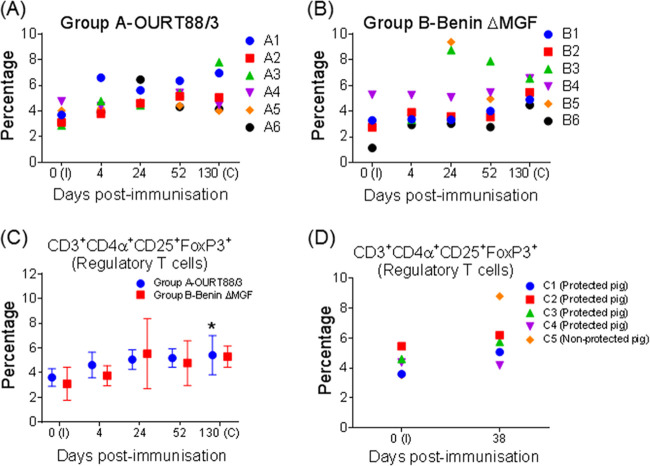
(A and B) Percentages and kinetics of Tregs (CD3^+^ CD4α^+^ CD25^+^ FoxP3^+^) in PBMCs collected from pigs at different days after immunization with OURT88/3 (A) or BeninΔMGF (B). (C) Changes in regulatory T cells (Tregs) after immunization with OURT88/3 or BeninΔMGF. Results show the percentages (mean ± SD) and kinetics of Tregs (CD3^+^ CD4α^+^ CD25^+^ FoxP3^+^), assessed by flow cytometry. Black asterisks indicate statistically significant differences (one-way analysis of variance) between preimmunization percentages at day 0 p.i. and prechallenge percentages at day 130 p.i. within each experimental group (*, *P* < 0.05). I, immunization day; C, challenge day (*x* axis). (D) Percentages and kinetics of Tregs (CD3^+^ CD4α^+^ CD25^+^ FoxP3^+^) in frozen PBMCs collected over the course of previous independent experiments (24) from protected and unprotected pigs immunized intramuscularly with 10^4^ TCID_50_ of BeninΔMGF and challenged with 10^4^ TCID_50_ of ASFV isolate Benin 97/1. The frozen PBMCs analyzed were taken at days 0 and 38 postimmunization.

Regulatory T cells (Tregs) may have interfered in generating a specific immune response against ASFV in the immunized pigs included in the present study. To assess this hypothesis, CD3^+^ CD4α^+^ CD25^+^ FoxP3^+^ cells in PBMCs from pigs immunized with OURT88/3 (group A) or BeninΔMGF (group B) were analyzed at different times postimmunization and before challenge (days 0, 4, 24, 52, and 130 p.i.). Tregs exhibited a progressive increase in both groups of immunized animals, peaking at day 130 p.i., just before challenge, and showing statistically significant differences from the preimmunization values (Fig. 9A to C).

Therefore, the increase in Tregs might represent a viral strategy to prevent immune responses against ASFV. To test whether such an increase was an isolated event or might represent a more general feature in cases in which protection was not achieved after ASFV infection, frozen PBMCs collected in the course of previous independent experiments from protected and unprotected pigs (24) were also analyzed. These pigs (*n* = 5) were immunized intramuscularly with 10^4^ TCID_50_ of BeninΔMGF, boosted at day 21 postimmunization with the same dose and by the same route, and finally, challenged intramuscularly 18 days after the boost with 10^4^ TCID_50_ of the parental virulent ASFV isolate Benin 97/1. The frozen PBMCs analyzed were taken in anticipation of future immunological studies before immunization and after the boost (day 0 and 38 p.i., respectively). As shown in Fig. 9D, Tregs exhibited an increase in percentage at day 38 p.i. only in unprotected pig C5, whereas the four protected animals (pigs C1 to C4) did not show an increase and had similar percentages of Tregs. Despite differences in the experimental design and regardless of whether the animals had received a boost vaccination or not, these results suggest that the changes in the Treg population observed at day 130 p.i. in the present experiment might correspond with a putative role in suppressing protective immune responses against ASFV.

### FoxP3 regulatory T cell ability to inhibit proliferative responses.

**FIG 10:**
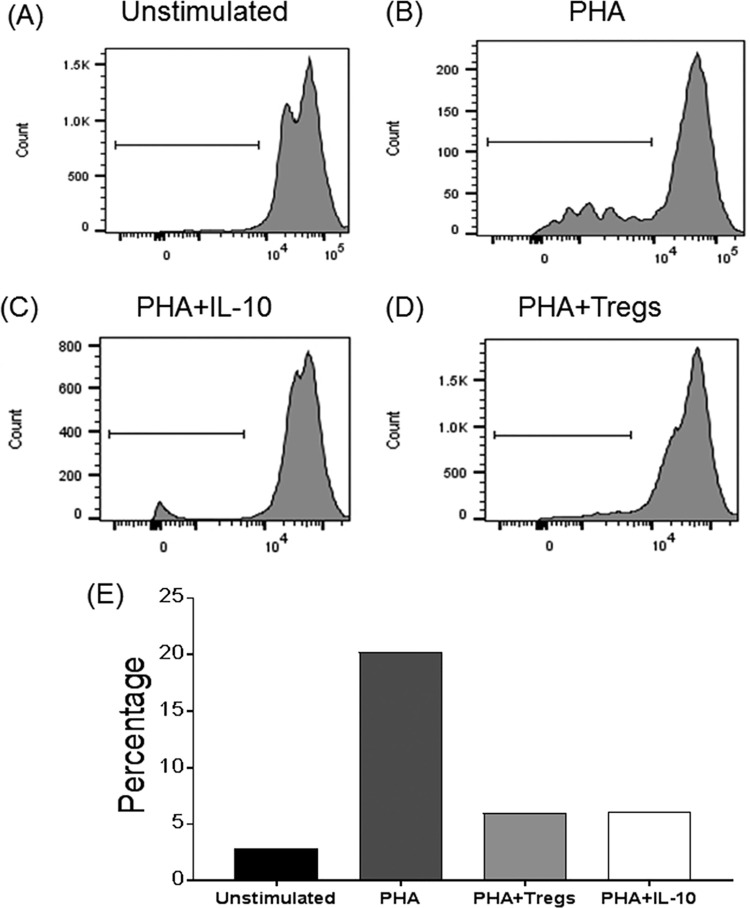
Suppressive activity of regulatory T cells (Tregs) sorted from pigs immunized with OURT88/3 (data not shown) or BeninΔMGF on autologous lymphocytes. (A) Unstimulated lymphocytes. (B) Proliferating lymphocytes after PHA stimulation. (C) Proliferating lymphocytes after PHA stimulation in the presence of IL-10. (D) Proliferating lymphocytes after PHA stimulation in the presence of sorted Tregs. (E) Summary of percentages of proliferating lymphocytes with the different stimuli. This is a representative experiment out of three performed with cells from pigs A1, A3, B4, and B5.

A higher percentage of Tregs circulating in blood may represent an inhibition of specific immune responses at the time of challenge. To test this hypothesis, sorted Tregs from vaccinated animal PBMCs were tested for their ability to inhibit proliferative responses *in vitro*. PBMCs from the same individual at day 0 were left unstimulated (Fig. 10A) and stimulated with PHA in the presence or not of IL-10, which is capable of inhibiting proliferative immune responses (Fig. 10B and C). Likewise, sorted CD4α^+^ CD25^high^ T cells (Fig. 10D) were added to PHA-stimulated cultures to test their putative inhibitory responses. As shown in Fig. 10E, CD4α^+^ CD25^high^ T cells from pig B4 immunized with BeninΔMGF (group B) exhibited an ability to inhibit proliferative responses to a certain extent when incubated *in vitro* with PHA-activated PBMCs.

### Evaluation of IL-10 levels in serum samples.

The levels of IL-10 in serum samples taken before and after immunization (day 0, 4, and 24 p.i.), after challenge (day 3 p.c.), and at termination were analyzed by ELISA. After immunization, no significant changes in IL-10 concentrations in pigs immunized with OURT88/3 or BeninΔMGF were observed (Fig. 5L). High levels of IL-10 were detected in both groups of immunized pigs after challenge. All immunized pigs showed an increase in IL-10 concentrations just before euthanasia (Fig. 5I and J). The levels were especially high in some pigs that were immunized with BeninΔMGF and that were euthanized at early dates (pigs B2, B3, and B6), although statistical analysis did not show significant differences at termination for the group of pigs immunized with OURT88/3 or the control group (Fig. 5K).

## DISCUSSION

Following short immunization protocols, attenuated viruses OURT88/3 and BeninΔMGF have previously been shown to induce a high percentage of protection against challenge with virulent virus that ranged from 70 to 100% (15, 22–24). Here, we tested whether a single dose of these viruses could induce a longer-term protection against challenge. Clinicopathological, virological, and immunological parameters were also evaluated over an extended period of 130 days in domestic pigs. Our results showed that a single intramuscular immunization followed by a challenge with the virulent Benin 97/1 ASFV genotype I isolate at day 130 postimmunization did not induce long-term protection against disease. All pigs developed acute forms of ASF and were euthanized at a moderate-severity humane endpoint.

Compared to the genome sequences of virulent isolates, including Benin 97/1, the naturally attenuated isolate OURT88/3 has deletions from a genome region encoding members of the multigene families MGF 360 and MGF 530/505 (25), whose function has been associated with the inhibition of type I IFN induction and responses in infected cells (20, 22, 27, 28). Deletion of these genes from the virulent isolate Benin 97/1 reduced virulence and produced a promising attenuated live vaccine candidate, BeninΔMGF (22, 24). Type I IFNs (IFN-α/β) play a key role in the activation of the innate immune response early in viral diseases and in controlling viral replication (29), the maturation of conventional dendritic cells (DCs), and the cross-presentation of viral antigens to CD8^+^ cells as well as in the activation of NK cells (30, 31). In the present study, a mild but significant increase in IFN-α serum levels was detected in the group of pigs immunized with BeninΔMGF at day 4 p.i. and after challenge, just before euthanasia of the pigs. Such changes were not detected in most of the pigs immunized with OURT88/3, in agreement with previous results (28). In contrast, nonimmunized control pigs displayed an increase in IFN-α levels in serum to concentrations higher than those detected in immunized pigs after challenge. A similar increase was described in pigs infected with other virulent ASFV isolates (28, 32). However, high levels of virus replication were detected at this time, suggesting that IFN-α was induced too late to control virus replication.

Plasmacytoid dendritic cells (pDCs) are recognized as specialist type I IFN-producing cells (28, 33). However, in the present study, the percentages of these cells increased only moderately at day 52 p.i., and they showed low percentages before the challenge (day 130 p.i.) in both groups of immunized pigs. NK cells are considered to play a major role in combating viral infections by directly killing virus-infected cells. The low percentages of NK cells detected before challenge combined with the low percentage of pDCs (34) may have interfered with the activation of an effective innate immune response to control virus replication.

In the course of viral infections, IFN-γ, the only member of the so-called type II IFN, is secreted by activated NK cells and T lymphocytes, dendritic cells, and macrophages, playing an important role in stimulating a cell-mediated immune response (29, 35, 36). As in previous experiments (22–24), early high levels of IFN-γ were detected by ELISA at day 4 p.i. in serum samples from pigs vaccinated with BeninΔMGF, coinciding with virus replication in blood, but not in pigs immunized with OURT88/3, which had no detectable virus in blood. Furthermore, in both groups of vaccinated pigs, stimulation of PMBCs with BeninΔMGF induced an increase in the levels of IFN-γ-secreting cells detected by ELISpot assay, with higher levels being induced by stimulation with BeninΔMGF than by stimulation with OURT88/3 or Benin 97/1. This may reflect differences in the efficiency of antigen presentation in cells infected with these different isolates. Despite the differences in experimental design, the levels of ASFV-specific IFN-γ-producing cells detected by ELISpot assay were similar to those detected previously in pigs which survived challenge at equivalent dates after immunization with OURT88/3 or BeninΔMGF (15, 22). A similar correlation was detected in some studies with different vaccine candidates (26, 37–42) but not in others (16, 43–45); thus, the induction of IFN-γ-specific T cells was not considered a reliable indicator of protection (46). In the current study, the numbers of specific IFN-γ-producing cells detected by ELISpot assay decreased from a peak at day 24 p.i. in both vaccinated groups but were markedly elevated before challenge (day 130 p.i.). However, the fatal outcome of the experiment after challenge agrees with the fact that prechallenge high levels of ASFV-specific IFN-γ-producing cells do not guarantee protection against ASFV challenge.

While previous *in vivo* immunization studies against ASFV had speculated that possible sources of IFN-γ are T-cell subsets (26, 37–40), in the present *in vivo* study, the phenotype of the IFN-γ-producing cells was fully characterized. The percentages of γδ-T cells, T-helper cells (CD4^+^), and double-negative T cells secreting IFN-γ were significantly higher in the group of pigs immunized with BeninΔMGF than in the group of pigs immunized with OURT88/3 at day 24 p.i., when these cell subsets peaked. The percentages of these cells decreased significantly after this day. These differences, along with the differences in IFN-α and IFN-γ levels discussed above, may have contributed to a slight delay in the fatal outcome after challenge described in pigs vaccinated with BeninΔMGF. In both groups of pigs, a significant transient increase, which peaked at day 24 p.i., was followed by a sharp decrease, which reached preimmunization values at day 130 p.i., in the percentages of NK cells, cytotoxic T cells (CD8^+^), and, particularly, double-positive cells (memory cells) secreting IFN-γ. In PBMCs, the percentages of antigen-presenting cells (monocytes and dendritic cells) and T-cell subsets (T-helper cells CD4^+^, cytotoxic T cells [CD8^+^], γδ-T cells, and double-positive T cells) seen at day 130 p.i. were similar to those seen preimmunization. Thus, the results provide evidence that one single immunization of pigs with the vaccine candidates tested in the present study was not enough to maintain the level of cellular components and antiviral chemical mediators required to induce a lasting immune response.

However, despite the fatal outcomes that occurred in the present experiment, we cannot rule out the possibility that the high levels of IFN-γ-secreting cells detected by ELISpot assay, along with the significant increases in specific T-cell subsets secreting IFN-γ at day 24 p.i. in both immunized groups, might explain the high percentages of protection described in previous studies, in which domestic pigs were challenged with virulent ASFV isolates 3 weeks after immunization or a boost with BeninΔMGF or OURT88/3 (15, 22–24). In view of the results obtained, a boost between days 24 and 52 p.i. could probably have contributed to the maintenance of or an increase in the level of T-cell subsets secreting IFN-γ or triggered the immunological mechanisms responsible for long-term protection in pigs. However, both how many boosts are required to induce a lasting protection and the intervals at which they should be administered are questions that remain unanswered and that future studies should address.

Taken together, these results suggest that in the current study (i) the levels of IFN-γ-secreting cells detected by ELISpot assay were not a good predictive biological parameter to evaluate the long-term protection induced; (ii) the prechallenge percentages of cellular components identified as the source of IFN-γ were not high enough to trigger an immune response capable of protecting pigs against ASFV; and (iii) the decrease in the percentages of these cellular components in PBMCs suggests an important role for CD8^+^ cells in protection, as previously suggested (11, 22, 37–40, 45, 47). Following this line of thought, our results suggest a possible role in protection for different CD8^+^ subsets secreting IFN-γ. Thus, along with the cytotoxic T cells, other cellular populations, such as double-positive T cells (defined as memory T cells) which display swine leukocyte antigen I [SLA-I]-restricted cytotoxic activity (34, 48), NK cells (whose increased activity was described in protected pigs previously immunized with a naturally attenuated ASFV isolate [17, 49, 50]), and γδ-T cells (a cell subpopulation able to present ASFV antigens to specific T cells and secrete IFN-γ [51]), should be highlighted as having a possible role in ASFV protection mechanisms. Recently, porcine invariant natural killer T-cell (iNKT-cell) functional subsets have been defined in pigs, and their capacity to secrete IFN-γ after activation was confirmed (52). After ASFV Armenia08 infection, the iNKT-cell frequency increased in peripheral blood, although no detectable activation of iNKT cells was observed after ASFV infection (52). Most of the iNKT cells in our experiments would have been located within the double-negative subset of T cells (CD3^+^ CD4α^−^ CD8α^−^) or in the CD8^+^ T-cell subset (CD3^+^ CD4α^−^ CD8α^+^) and might have constituted a major part of the IFN-γ-secreting cells in those subsets. In addition, a decrease in the percentages of T-helper CD4^+^ cells might also have compromised activation of the protective cellular immune response.

Regulatory T cells produce multiple secretory inhibitory cytokines, such as transforming growth factor β (TGF-β) or IL-10, which play a prominent role in Treg-mediated suppression (53). Relevant activities of IL-10 include inhibition of NK cells and the adaptive cellular immune response; downregulation of major histocompatibility complex class II (MHC-II) expression; and suppression of proinflammatory cytokines involved in several immune functions, such as the growth of different cell types, activation of macrophages, antibody production, and chemotactic mechanisms (54). Certain viruses have evolved immunosuppressive strategies by encoding homologues of anti-inflammatory cytokines, including IL-10 and TGF-β, capable of preventing the induction or activation of cytotoxic T-lymphocyte responses (55). In the case of ASFV, it is plausible to speculate that several cellular subsets might be involved in an aberrant immune response in which IL-10 plays a central role, as it has been shown in bacterial infections (56). In this sense, previous *in vitro* studies demonstrated an inhibition of proinflammatory cytokine expression and increased expression of anti-inflammatory cytokines on porcine macrophages infected with virulent ASFV isolates (57–59).

Tregs account for an increased virus load by restraining IFN-γ responses from virus-specific CD8^+^ T cells in the mouse model of herpes simplex virus (HSV), and subsequent reports demonstrated a role for Tregs in viral persistence and blunting the immune response to other viruses, including HIV, hepatitis B virus (HBV), and hepatitis C virus (HCV) (60). Different studies have reported that in the course of porcine viral infections, virus-induced Tregs produce IL-10 and TGF-β, a mechanism that allows viruses to persist in the host by controlling the intensity of IFN-γ production and cytotoxic activity (61). On the other hand, recent studies have reported a decrease in the percentage of Treg-positive cells in blood within 5 days postinfection with a moderately virulent ASFV isolate (62), whereas the results obtained in the present study show an increase in Tregs during a prolonged period of time after immunization with attenuated ASFV isolates. Furthermore, those Tregs were functional, as they were able to suppress the PHA-induced proliferation of cells when placed into culture. The level of inhibition was similar to the one induced when IL-10 was added in the culture. Thus, it is plausible to speculate that Tregs might be responsible for inhibiting the proliferation of ASFV antigen-specific cells capable of controlling viral infection in the animal.

Although the source of IL-10 was not determined, in the course of the present experiment, Tregs exhibited a progressive increase in both groups of immunized animals, peaking at day 130 p.i., just before challenge, and showing statistically significant differences from the preimmunization values. As in previous *in vivo* experiments in which domestic pigs were immunized with BeninΔMGF or OURT88/3 (22–24), all immunized pigs that did not become protected showed an increase in IL-10 serum concentrations just before euthanasia. In a previous short-term study, it was suggested that γδ-T cells and IL-10 levels could be related to mortality (62). However, these new results suggest that both Tregs and IL-10 may contribute to the ASFV strategy to interfere with the generation of a specific immune response by controlling antiviral IFN levels and the cell-mediated immune response. Thus, it is tentative to suggest that high levels of IL-10, together with increased numbers of Tregs, could be related to a lack of ASFV control by the immune system. In order to improve vaccine efficacy, further knowledge of Treg immunoregulatory mechanisms and the role of anti-inflammatory cytokines in the course of immunization against ASFV would be required.

## MATERIALS AND METHODS

### Viruses.

The low-virulence nonhemabsorbing ASFV isolate OURT88/3, the attenuated deletion mutant BeninΔMGF (obtained from virulent isolate Benin 97/1), and the virulent isolate Benin 97/1, all belonging to genotype I, were previously reported (15, 22, 25). Virus stocks were grown in porcine bone marrow (PBM) cell cultures, and supernatants from cultures were harvested and titrated by limiting dilution in PBM cell cultures. Infected cells were detected by immunofluorescence using a monoclonal antibody (MAb) against ASFV protein p30. Virus titers are presented as the amount of virus infecting 50% of the macrophage cultures (numbers of TCID_50_ per milliliter).

### Immunization, challenge, sampling, and clinical evaluation of pigs.

Experiments were conducted in high-containment facilities at The Pirbright Institute and regulated by the Animals (Scientific Procedures) Act UK 1986 (Home Office project license 70/7725). Large White and Landrace crossbred female pigs, 9 to 10 weeks old (weight, 21 to 25 kg), from a high-health-status herd were used. Pigs were separated into two groups of six pigs each. Group A animals (pigs A1 to A6) and group B animals (pigs B1 to B6) were immunized by the intramuscular route in the neck muscles with 1 ml containing 10^4^ TCID_50_ of OURT88/3 isolate (group A) or the same titer of BeninΔMGF (group B). At day 130 postimmunization (p.i.), all immunized pigs, in parallel with three control nonimmune pigs of the same age, which were introduced in a separate room before challenge (pigs C1 to C3), were challenged intramuscularly with 1 ml containing 10^4^ TCID_50_ of the genotype I virulent ASFV isolate Benin 97/1. After the challenge, the pigs were euthanized at different time points, once animals reached a moderate-severity humane endpoint. After immunization and challenge, the viruses were retitrated using the remaining inoculum.

The immunization day was defined as day 0 (day 0 p.i.). EDTA-anticoagulated blood and serum samples were collected longitudinally over the course of the experiment prior to virus immunization (day 0 p.i.), after immunization (at days 4, 14, 24, 52, 89, and 130 p.i.), at day 3 postchallenge (p.c.), and at different termination days. These samples were stored (−80°C).

Rectal temperatures and clinical signs were monitored as previously described (15). Data were recorded daily up to day 21 p.i. Subsequently, data were taken twice per week up to the challenge day (130 p.i.). After challenge, clinical evaluations were carried out daily until the end of the experiment. Macroscopic lesions were evaluated during necropsies, using scores based on previous standardized protocols (63).

### ASFV detection in blood and evaluation of antibodies and cytokines in serum samples.

DNA was extracted from the EDTA-anticoagulated blood samples using a MagMAX system (Thermo Fisher) and a Magvet universal isolation kit (Life Technologies). qPCR was carried out following a modified protocol described previously (15, 64). Infectious virus in blood was titrated by endpoint dilution in PBM cells. Infected cells were detected by immunofluorescence using a monoclonal antibody against the ASFV p30/CP204L protein.

Serum samples were assayed using a commercial competition ELISA kit for the detection of ASFV-specific antibodies against VP72 (Ingezim PPA3 Compac; Ingenasa, Madrid, Spain) and by commercial ELISA kits for the detection of porcine immunoregulatory cytokines (IFN-γ and IL-10; R&D Systems, Abingdon, UK) according to the manufacturers’ instructions. For IFN-α, an in-house ELISA using antibodies purchased from PBL Interferon Source were used as previously described (65).

### Purification and cryopreservation of PBMCs.

Fresh EDTA-anticoagulated blood samples collected from all vaccinated pigs prior to virus immunization (day 0 p.i.) and after immunization (at days 4, 24, 52, 89, and 130 p.i.) were used to isolate peripheral blood mononuclear cells (PBMCs). Fresh EDTA-anticoagulated blood samples were also taken from nonimmunized control pigs (pigs C1 to C3) before challenge (at day 130 p.i.), at day 3 p.c., and at termination (day 5 p.c.). PBMCs were obtained by density gradient centrifugation and used fresh for phenotyping/functional analysis or cryopreserved for subsequent analysis as previously described (66).

### Immunophenotyping of PBMCs by flow cytometry.

Fresh isolated PBMCs were suspended, and cell densities were adjusted. Then, 1 × 10^6^ cells contained in 50 μl were transferred to the wells of a 96-well round-bottom microtiter plate (Costar; Fisher Scientific, Leicestershire, UK) and incubated with 10% normal goat serum in phosphate-buffered saline (PBS) for 10 min at room temperature (RT) (diluted 1:1). After incubation, the cells were washed twice with PBS–0.1% bovine serum albumin (BSA) and stained in duplicate wells with different combinations of MAbs specific for surface markers (50 μl/well of an MAb cocktail) for 10 min at RT and then washed twice. The conjugated MAbs used were anti-CD14 (phycoerythrin [PE]; clone TUK4; Miltenyi Biotec, Bisley, UK), anti-pig CD3e (PE-Cy7; clone BB23-8E6-8C8; BD Biosciences), anti-pig CD4α (peridinin chlorophyll protein-Cy5.5; clone 74-12-4; BD Biosciences), and anti-pig CD8α (fluorescein isothiocyanate; clone 76-2-11; BD Biosciences). The nonconjugated MAbs anti-pig CD25 (clone K231.3B2; Bio-Rad, Kidlington, UK) and anti-CD172a (clone BA1C11; INIA, Madrid, Spain) were labeled using a Zenon Alexa Fluor 488 mouse IgG1 labeling kit according to the manufacturer’s instructions (Thermo Fisher), while the MAb swine TCR1 delta chain (clone PGBL22A; Kingfisher, Saint Paul, MN, USA) and swine MHC-II (clone MSA3; Kingfisher, Saint Paul, MN, USA) were labeled using a Lightning-Link PE-Texas Red tandem conjugation kit according to the manufacturer’s instructions (Innova Biosciences, Cambridge, UK). Cell viability was assessed by staining with a LIVE/DEAD Fixable Aqua dead cell stain kit (Thermo Fisher). Surface-stained cells were fixed with 50 μl/well of 4% paraformaldehyde for 30 min at RT. Then, the cells were washed twice and resuspended in PBS–0.1% BSA prior to flow cytometric analysis. An IgG1-Zenon Alexa Fluor 488 isotype control MAb (INIA, Madrid, Spain) and an IgG2a-PE-Texas Red isotype control MAb (Kingfisher, Saint Paul, MN, USA) were used to control staining with anti-CD172a and swine MHC-II MAbs, respectively. Unstained cells were used as a control with the other antibodies.

For intracellular IFN-γ staining, fixed surface-stained cells were permeabilized with Cytofix/Cytoperm solution (BD Bioscience) for 30 min at 4°C in the dark. After two washes in BD Perm/Wash buffer (BD Biosciences), the PBMCs were incubated with the MAb anti-pig IFN-γ (PE; clone P2G10; BD Biosciences) at 4°C in the dark for 30 min (50 μl μl/well). Then, the cells were given two final washes in BD Perm/Wash buffer and resuspended in PBS–0.1% BSA prior to flow cytometric analysis. An IgG1-PE isotype control MAb (Kingfisher, Saint Paul, MN, USA) was used for control staining with an IFN-γ MAb. In order to evaluate FoxP3 intracellular expression, fixed surface-stained cells were permeabilized with Fixation/Permeabilization working solution, prepared according to the manufacturer’s instructions (Affymetrix eBioscience), for 30 to 60 min at RT in the dark. After two washes in permeabilization buffer (Affymetrix eBioscience), PBMCs were incubated with the MAb anti-mouse/rat FoxP3 (allophycocyanin [APC]; FJK-16s; Affymetrix eBioscience) at RT in the dark for 30 min (50 μl/well). Finally, the cells were washed twice in permeabilization buffer and resuspended in PBS–0.1% BSA prior to flow cytometric analysis. A rat IgG2a-APC isotype control MAb (Affymetrix eBioscience) was used as a control for staining with FoxP3 MAb.

**FIG 11:**
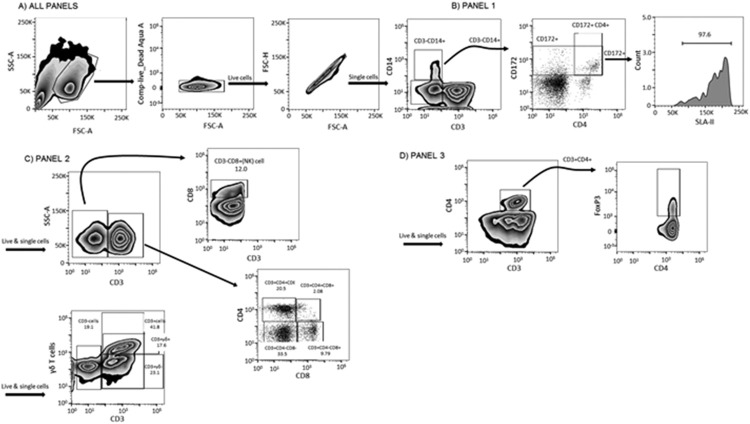
Gating strategies applied to data from flow cytometry. (A) All cells were gated by their side scatter and forward scatter and live/dead gate, and, finally, doublets were removed. The following analysis was done only with live single cells. (B) Gating strategy for panel 1. (C) Gating strategies for panel 2. (D) Gating strategy for panel 3. SSC-A, side scatter area; FSC-A, forward scatter area.

Three panels including different combinations of the antibodies described above were used. LIVE/DEAD Fixable Aqua dead cell stain was used in all panels to exclude dead cells. In panel 1, the combination of anti-CD3, CD4α, CD14, CD172, and SLA-II was used for identification of antigen-presenting cells (monocytes, CD3^−^ CD14^+^; conventional dendritic cells, CD3^−^ CD14^−^ CD172^+^ SLA-II^+^; plasmacytoid dendritic cells, CD3^−^ CD14^−^ CD172^+^ CD4α^+^ SLA-II^+^). In panel 2, T cell populations secreting IFN-γ were distinguished through the expression of CD3, CD4α, CD8α, TCRγδ, and IFN-γ (cytotoxic T cells, CD3^+^ CD4α**^−^** CD8α^+^; γδ-T cells, CD3^+^ CD4α**^−^** TCRγδ^+^; T-helper cells, CD3^+^ CD4α^+^ CD8α^−^; double-positive T cells, CD3^+^ CD4α^+^ CD8α^+^; natural killer cells, CD3^−^ CD4α^−^ CD8α^+^; double-negative T cells, CD3^+^ CD4α^−^ CD8α^−^). In panel 3, the combination of CD3, CD4α, CD25, and FoxP3 was used for the identification of regulatory T-cell (Treg; CD3^+^ CD4α^+^ CD25^+^ FoxP3^+^) populations. Cells were analyzed using a BD LSRFortessa flow cytometer. In each gating strategy, about 10,000 cells were collected in the selected gate. The percentages of positive cells within a gate were considered within the same gate and not from the total population of cells. The gating strategy is shown in Fig. 11. Data were analyzed using FlowJo software (v10; Tree Star, Ashland, OR, USA).

### Stimulation of specific T-cell IFN-γ responses.

The cell densities of fresh isolated PBMCs were adjusted. Then, 1 × 10^6^ cells contained in 100 μl were transferred to the wells of a 96-well round-bottom microtiter plate (Costar; Fisher Scientific, Leicestershire, UK). The PBMCs were stimulated in duplicate wells with 50 μl of ASFV isolate OURT88/3, BeninΔMGF, or Benin 97/1 at a multiplicity of infection of 0.5. Mock-stimulated wells were used as negative controls, while wells stimulated with 0.2-μg/ml phorbol 12-myristate 13-acetate (PMA) and 0.2 μg/ml ionomycin (ION) (Sigma-Aldrich, St. Louis, MO, USA) were used as positive controls. Cells were incubated overnight (37°C, 5% CO_2_ for 14 to 15 h). Then, brefeldin A (GolgiPlug; BD Biosciences) was added (1 μl/ml) and the cells were further incubated for 6 h. Subsequently, the cells were washed twice with PBS–0.1% BSA and then incubated with 10% normal goat serum in PBS for 10 min at RT. To detect T-cell populations secreting interferon, cells were stained with MAbs (CD3, CD4α, CD8α, TCRγδ, and IFN-γ) following the protocols described above for surface markers and intracellular IFN-γ secretion.

### IFN-γ ELISpot assay.

To determine the number of antigen-specific IFN-γ-secreting cells, IFN-γ ELISpot assays were carried out as previously described (67). Briefly, PBMCs purified from EDTA-anticoagulated blood by gradient centrifugation were plated in triplicate at a dilution of 5 × 10^5^ cells per well. They were then incubated overnight in a final volume of 100 μl with ASFV isolate Benin 97/1, BeninΔMGF, or OURT88/3 with 1 × 10^5^ 50% hemadsorption doses of virus stocks or an equivalent volume of mock inoculum and 2.5-μg/ml phytohemagglutinin (PHA) as a positive control. Biotinylated anti-swine IFN-γ MAb (P2C11; Thermo Fisher), followed by streptavidin conjugated to alkaline phosphatase, was used to visualize the spots, which were then counted using an ELISpot assay reader system (Autoimmun Diagnostika GmbH, Strassberg, Germany). The mean number of spots from triplicate wells was plotted and is represented as the number of IFN-γ-producing cells per 10^6^ cells after subtraction of the average number of IFN-γ-positive cells in mock-inoculated control wells.

### Proliferation assays.

Frozen PBMCs from animals A3, B4, and B5 were used in proliferation assays. Cells collected from the same animal at day 0 (uninfected) and at day 24 postvaccination were thawed in each assay. Day 0 cells were stained with Cell Trace Violet following the instructions from the proliferation kit (catalog number C34557; Invitrogen). Day 24 cells were depleted of B cells (clone CC5 1-6; The Pirbright Institute, UK), monocytes (clone CCg33 1-3; The Pirbright Institute, UK), and CD8-positive cells (clone 11-295-33; The Pirbright Institute, UK). They were all supernatants that had previously been titrated and were used at a 1:10 dilution as the saturating concentration. Cells were washed in cold PBS–2 mM EDTA, and anti-mouse IgG magnetic beads (Miltenyi Biotec, Bisley, UK) were added following the manufacturer’s instructions. Flowthrough cells were further stained with anti-CD4α and anti-CD25 MAbs as stated above and sorted in a BD FACSAria III cell sorter. CD4α^+^ CD25^high^ cells were selected as Tregs. CD4α^+^ CD25^−^ cells were selected as controls. Cells were cultured in 96-well round-bottom plates with 10^4^ day 0 cells with or without stimuli or cocultured with 1,500 to 2,000 day 24 sorted cells per well, as stated above. Phytohemagglutinin (PHA; Sigma-Aldrich, St. Louis, MO, USA) was used at 1.25 μg/ml per well, and recombinant porcine IL-10 (R&D Systems, Abingdon, UK) was used at 12.5 pg/ml per well. The plates were left in the incubator for 5 days, and then the cells were fixed with 4% paraformaldehyde and run in a BD LSRFortessa flow cytometer.

### Statistical analysis.

GraphPad Prism (v7.0) software (GraphPad Software, La Jolla, CA, USA) was used for graphical and statistical analysis of the data sets. The statistical tests applied to each data set are indicated in the figure legends.
